# A critical analysis of the networking experiences of female entrepreneurs: a study based on the small business tourism sector in Sri Lanka

**DOI:** 10.1186/s13731-022-00255-y

**Published:** 2022-11-25

**Authors:** H. A. K. N. S. Surangi

**Affiliations:** Department of Commerce and Financial Management, Faculty of Commerce and Management Studies, University of Kelaniya, Kelaniya, Sri Lanka

**Keywords:** Female entrepreneurs, Networking, Social constructionist, Narratives, Tourism

## Abstract

This study expands on current knowledge through how female entrepreneurs form and develop their networks in the Sri Lankan context. It adopts social constructionism philosophy and narrative design to explore the female entrepreneurs' networking behaviour. Thematic analysis is used to understand the life stories of fourteen women entrepreneurs in the tourism sector. Findings suggest that female entrepreneurs are likely to rely on more informal recruitment methods and informal training practices. They have strong relationships with local communities, but they focus on customers beyond the locals. Seasonality within tourism has emphasised tourism literature due to its disruptive effect on economic transactions. However, less of the literature has examined the social effects of seasonality, which is where this study can contribute by exploring how gender roles related to social and domestic responsibilities are renegotiated during the low and high seasons when tourism entrepreneurs re-adjust to new time-demand realities. Nevertheless, the narrative research design is not widely used in the Sri Lankan context. Therefore, this article adds to the entrepreneurial networking knowledge by analysing stories about female entrepreneurs' experiences and social constructionist perspectives.

## Introduction

Research focusing on female entrepreneurs in other socio-cultural contexts is relatively new and limited in number (Arasti et al., 2021; Kirkwood, 2012; Roomi et al., 2009). While gender is a heavily researched area, however, on the other hand, female entrepreneurship and networking as one are rarely considered when examining the tourism phenomena (Figueroa-Domecq, et al., 2015; Valeri & Katsoni, 2021). This study contributes toward filling this gap by focusing on the networking experiences of female entrepreneurs in small businesses in the tourism sector in Sri Lanka. Furthermore, this research demonstrates that female entrepreneurs in Sri Lanka face different constraints and opportunities from developed countries, either in absolute or relative terms, and these might make their networking experiences different from female entrepreneurs in developed countries.

Throughout this article, the author uses the term entrepreneurs’ networks; networks can be defined as entrepreneurs’ relationships or connections with external parties (Bhushan et al., 2020; Premaratna, 2002). The external parties would be individuals or organisations. Furthermore, the author uses the verb ‘to network’ and the participle form ‘networking’, both meaning the actions by which an entrepreneur creates and develops contacts. Entrepreneurs form and develop network relationships to acquire required resources and identify business opportunities (Arasti et al., 2021; Valeri & Baggio, 2021b). Those who perform and control the resources are called network actors; they can be informal or formal organisations (Valeri & Baggio, 2021a, 2021b). According to the nature and objectives of the firm, network actors can be divided into four categories: social network, inter-firm network, supporting network, and business network actors. Social networks of contacts are made informally through social or non-business activities. These contacts may comprise family, relatives, friends, and acquaintances. Inter-firm networks of actors consist of other firms, for example, small, medium and large companies. Supporting networks of actors consist of government bodies, private supporting organisations, NGOs, and banks. The supporting network actors’ primary purpose is to provide various types of support to small businesses. Finally, business networks may include membership in various professional bodies, attending seminars and trade fairs, etc.

Most of the small firms owned by women cannot achieve their goals by themselves (Premarathna, 2002). Therefore, network formation is essential for small and medium businesses to consider when aiming to achieve growth (Arasti et al., 2021; Valeri, 2016). In addition, small firms need support and resources from outside organisations (Valeri, 2016) and relatives and friends (Klyver, 2011). Consequently, researchers have argued that small businesses’ success and survival mainly depend on networks’ support. Social network theory describes the various relationships between people and focuses on understanding how relationships connecting individuals, groups, or organizations generate benefits and opportunities for human behaviour (Premarathna, 2002; Valeri & Baggio, 2021b). Some research findings reveal that small business entrepreneurs use networking to improve marketing activities and innovations (Robinson & Stubberud, 2009), meaning that networks are precious to small businesses.

There is a strong relationship between women and the tourism sector. Women’s participation in entrepreneurial activities in the tourism sector is high compared to other industries (Figueroa-Domecq et al., 2015). For example, Latin America has the highest proportion of female employers in tourism, more than double the rate in other sectors (UNWTO, 2011). In Sri Lanka, finding information on gender participation in the tourism sector is somewhat challenging, as little information is available. Nevertheless, an unpublished report from the Department of Census and Statistics in Sri Lanka reveals that female entrepreneurs were particularly prevalent in the tourism sector (42%) compared to other industries (30%) in 2016. The tourism sector is characterised by seasonality and informality, and the majority of tourism employment is low-paid and part-time (Yunis, 2009).

Regarding the high number of women working in tourism, there is a relationship between this type of work and feminine subjectivities (Bakas, 2014). This circumstance makes one wonder if women’s presence within the tourism sector is a “chicken and egg” situation, whereby dominant gender structures push women into tourism, and whether, in turn, tourism encourages gendered labour (Bakas, 2014). However, a report (UNWTO, 2011) on the tasks women perform within the tourism and how tourism affects women reveals that tourism provides many opportunities for women to become employed, as there are low entry barriers, part-time work options, and everyday work, or home-based businesses.

The connection between the invisibility that women experience when completing domestic tasks and when working is significant, as this is internalized as a way of being and subsequently transcends the domestic boundary, contaminating women’s entrepreneurial intentions. In Bakas’s (2014) study, many of the female entrepreneurs in Italy show signs of how their natural role as domestic careers affects their interpretations of entrepreneurship by, for example, projecting how significant it is for them to help their family by becoming involved in entrepreneurship. The importance of building networks, relationships, exchanging resources, knowledge, mutual support, and developing trust in tourism activities has been emphasised in many studies (Valeri & Baggio, 2021b). For example, during the COVID 19 pandemic, Valeri and Baggio (2021b) uncovered that, for the survival of tourism-related businesses, an effective mechanism for knowledge sharing is essential. Furthermore, building a networking relationship for women to be active in the tourism sector is consistently highlighted in the literature. As women are not only faced with the challenges of dealing with economic activities but often also with the challenges of shifting their traditional gender roles in their communities by making incomes, becoming “businesswomen” in the tourism sector,

The tourism sector is dominated by small- and medium-sized enterprises as well as female businesses. Both groups usually do not have the resources to generate new knowledge, so they are forced to rely on external sources. In reality, among the various economic fields, tourism is where formal and informal cooperation, partnership and network are essential for the survival of a business (Valeri & Baggio, 2021a, 2021b). Researchers discovered that a lack of networks is the underlying reason why female entrepreneurs experience less success compared to their male counterparts (Wang et al., 2021). Therefore, there is essential to identify women’s networking experiences and networking strategies to meet these challenges. In this setting, the study seeks to answer the research question: how do female entrepreneurs form and develop their networks in the small business tourism sector in Sri Lanka?

## Literature review

The systematic literature review was carried out to identify major arguments and debates circulating in female entrepreneurs’ networking area. This paper reviews the literature on female entrepreneurship, female entrepreneurs’ networks, and female entrepreneurs in the tourism sector for over 20 years, from 1999 to 2021. The review criteria was based on the PRISMA publication standards. Articles for the study were carefully chosen using two leading databases, Scopus and Web of Science (Centobelli et al., 2017). After screening the titles, abstracts, and full texts of the retrieved results, the researcher assessed the relevancy of the articles and chose only the most relevant studies which met the standards according to relevancy.

### Female entrepreneurship

Much entrepreneurship literature highlights a link between masculinity and entrepreneurship, concluding that women who want to be entrepreneurs must follow these ideals, as feminine ideals of passivity, collectivity, and caring are weaknesses in entrepreneurship theorising (Brush & Cooper, 2012; Kamberidou, 2020). More recent studies which focus on female entrepreneurship from a feminist approach underscore how different conceptualisations of entrepreneurship are viable, such as a recent study challenging women’s “underperformance myth” (Foss & Henry, 2021; Marlow & McAdam, 2013). Today, it can be seen that female participation in entrepreneurial activities is increasing gradually; however, female entrepreneurship is still under research area (Brush & Cooper, 2012). Henry et al. (2021) revealed that it is still vital to research why female entrepreneurship is still an under-researched area across cultures, communities, and contexts. Researchers in this area mainly focused on addressing women’s financial constraints, issues related to competing in a business and family, and gender differences in entrepreneurial characteristics (Kamberidou, 2020). In most cases, researchers reveal that women are complex, multi-tasking persons who contest traditional gender identities, and women’s subordinate role is consistently highlighted (Beriso, 2021; Foss & Henry, 2021). However, few researchers focus on softer issues, such as personal networks (Brush & Cooper, 2012). Relationships with other parties have become the most crucial support for female entrepreneurs in both established and aspiring businesses in all sectors (Henry et al, 2021). Similarly, literature on emerging entrepreneurial ecosystems emphasises the role of social networks and social capital as major elements in an operating ecosystem (McAdam et al., 2019).

### Female entrepreneurs networks

In current literature, the female entrepreneurial experience is described through an angle of networking, where the relational dimension that explains the networks’ content has more value (Valeri & Katsoni, 2021). Researchers have found that gender differences in entrepreneurs’ constraints in terms of forming and developing networks, and these differences may lead to different economic and social consequences in any particular society (Arasti et al., 2021, Sonfield & Lussier, 2005). Several studies have shown that women networks include more strong ties (Klyver, 2011; Valeri & Katsoni, 2021). However, their network composition and content are considered less powerful, less wealthy, and less heterogeneous than their male counterparts (D’Exelle & Holvoet, 2011). In networking literature, some findings revealed that both men and women have a strong desire to network with the same gender (Garcia & Carter, 2009; Valeri & Katsoni, 2021); contrastingly, others find that both genders need to have primarily men (Abraham, 2020; Arasti et al., 2021; Valeri & Katsoni, 2021). Therefore, the networking relationship would be more critical for women in the early stages of the business (Arasti et al., 2021; Garcia & Carter, 2009), giving emotional support and providing solutions to the particular problems that challenge women, such as business–family conflict or lack of acceptability of women being headed (Henry, 2021; Roomi et al., 2009). Furthermore, it can be seen that men hold positions of higher status and fewer family responsibilities in many social structures compared to female entrepreneurs (D’Exelle & Holvoet, 2011; McAdam et al., 2019). Female entrepreneurial networks are the creation of the close association of family and work. Family is consistently recognised as the main motivation for a business start-up (Valeri & Katsoni, 2021).

Moreover, other authors argue that an extensive support network can create conflicting demands on the socio-emotional attention of female business owners (Lindvert et al., 2017). In this setting, female entrepreneurs are more expected to struggle to meet the expectations of both those who compliment them for being successful and encourage them to be responsible mothers, spouses, and daughters simultaneously (Surangi, 2018). This norm can result in an inconsistency in women’s commitment to a venture, thereby negatively affecting their business growth (Lindvert et al., 2017). Therefore, some authors conclude that an extensive network may constitute a disadvantage for female entrepreneurs.

### Gender, tourism and networks

As tourism is “created of human networks” (Aitchison, 2001), female entrepreneurs’ network experience in the tourism sector is an exciting and under-researched area (Figueroa-Domecq et al., 2015). However, the role of gender plays in the tourism sector shreds of evidence, as women are more likely to enter the tourism sector due to its flexibility and suitability for them to be engaged without challenging social norms regarding women’s roles as housewives (Bakas, 2014; Rao et al., 2021). An example of women in tourism businesses that follow societal expectations related to feminine predispositions of care is women’s involvement in making craft products to sell to tourists, as is the case with Mexican weavers (Cohen, 2001) and Mayan craftswomen (Cone, 1995). As these women can work on their craft production at home, they are perceived as also being able to combine a business activity with childcare; thus, they conform to feminine subjectivities connected to care. In addition, some women convert part of their house into a guesthouse or restaurant for tourists, thereby simultaneously staying at home and engaging in business (Cieck et al., 2017). In effect, most women who engage in tourism undertake work that is a “natural” addition to their daily housekeeping, such as cleaning, cooking, and welcoming guests (Bakas, 2014).

Despite the potential that exists for female entrepreneurs in the tourism sector (Khatiwada & Silva, 2015), women are facing unique issues and challenges such as social and cultural norms than men in starting and running their businesses, as well as in accessing economic resources (Cieck et al., 2017). Female tourism entrepreneurs face unique problems, such as seasonality and long days, affecting how they negotiate the activities needed to continue domestic life daily and inter-generationally (Bakas, 2014; Fiona, 2017). Besides, the gender norms rooted in societies limit women’s participation in social and business activities (Bakas, 2014).

Therefore, it is essential to explore further the underlying grounds that determine gendered choices regarding participation in tourism activities. Gender forms part of the main problems addressed by Kinnaird and Hall (1994), who argue that constructions of gender relations are context-specific. Aitchison (2001), Kabil et al. (2022) and Khatiwada and Silva (2015) are examples of academics who critically review the role gender plays within tourism, adding to the body of literature that Kinnaird and Hall (1994) started. Aitchison’s (2001) focus on “socio-cultural relationships” follows the cultural trend within tourism studies, proposing a focus on both the material and the culture as the spaces, where gender identities and connections are re-worked. Pritchard (2008) argues that tourism research must address the social relations, such as gender, that strengthen tourism processes by connecting empirical examples to theory to support the call for more critical analyses of tourism. However, focusing more specifically on the role that gender plays in tourism research, few academics have embraced this subject, apart from Tucker’s (2003) longitudinal study on female entrepreneurs in Turkey.

Moreover, the literature reveals that women’s contribution to business and economic growth is almost certainly underestimated (Ghosh, 2020). Many women work in the informal sector, and their business activity is not reflected in national statistics, particularly in developing countries, such as Sri Lanka. In general, women's businesses have high failure rates and are less successful in financial performance than their male counterparts. Further female entrepreneurs engage in low-value sectors, such as tourism, handicraft, garment, and other service sectors. Furthermore, women face gender-specific obstacles when starting their ventures (Kabil et al., 2022). The literature relating to women and tourism has been reviewed and expanded. It is particularly relevant to examine how gender functions within tourism, as the tourism sector, is highly gendered, with women engaged in feminised positions, such as hosts, cleaners and cooks (Figueroa-Domecq et al., 2020; Kinnaird & Hall, 1994). However, relatively few researchers have examined this subject since Swain’s (1995) seminal article, which brought attention to how gender often shapes tourism’s processes. The majority of these studies focus on the economic impacts of seasonality yet nevertheless overlook the influences of seasonality on women’s domestic activities. Researchers from different academic disciplines have discussed the concept of networking, particularly those in Business, Economics, and Sociology. However, a general conceptual framework for investigating networking is still absent (Figueroa-Domecq et al., 2020). While the studies reviewed here have certainly been informative in terms of revealing the different ties involved in the female business and the structural dimension of networking, there is little qualitative information available (Valeri & Baggio, 2021a, 2021b) as to how the entrepreneurs form and develop their networks, and what they bring to the entrepreneurial venture through networks. Therefore, there remains an opening for theoretical and empirical research in the field of female entrepreneurs’ networks.

## Methodology

This study specifically focuses on female entrepreneurs’ networking relationships. However, these relationships do not exist within a vacuum and are part of wider societal structures. This emphasis on relationships and broader societal influences led the researcher to dig further into social constructionism's roots, considering it a possible philosophical grounding for this study. This is because social constructionism revolves around how people interpret what they perceive and how human activity creates that reality. The narrative approach is more appropriate for studying subjectivity and identity due to the importance of imagination and human involvement in constructing a story (Riessman, 2002). Therefore, narrative inquiry as a research design can be considered an integral part of producing new knowledge, allowing the depth of women’s experiences to be better understood in this study.

The study was conducted in Sri Lanka because of the researcher’s familiarity with and exposure to the Sri Lankan environment. Therefore, female entrepreneurs were selected who are involved in the tourism industry in the Southern Province, where most tourist arrivals occur and where many tourist activities are catered for. The purposive sampling method was used to choose study participants. Various strategies such as extreme or deviant case sampling, intensity sampling, great diversity, typical case sampling and opportunistic sampling were used to select information-rich cases. An extreme group or maximum heterogeneity approach produces an initial potential sample, and the final selection, then, had made a combination approach. The extreme or deviant case sampling approach emphasises cases rich in information, because they are unique and stand out. For example, the researcher chose Hemali as a respondent for this study as she was the best businessperson in the Southern Region in 2007 and the best businesswoman of 2008. The key informants in this study included, and these key informants mainly provided preliminary information about the research participants of the community and the general background of the area. In typical case sampling, participants were selected with the cooperation of key informants. An opportunistic sampling strategy was also used to choose research participants. For example, the researcher heard about one participant through another respondent during fieldwork; the researcher was told that her restaurant was the oldest one in the area, and she decided to choose her as a participant in the study.

The researcher looked for entrepreneurs with different backgrounds and unique stories to tell. Regarding routes to entrepreneurship, the researcher sought to find people coming out of unemployment, directly from the university or who had previously been working as an employee. One person of particular interest was Kumudu, who had left the public sector to become an entrepreneur. In line with Hytti (2005), since female tourism entrepreneurs were much debated at the end of the 1990s, the researcher sought to include representatives of the new wave‖ of entrepreneurs in this study. While most Sri Lankan entrepreneurs are currently middle-aged, entrepreneurs of all age groups were needed for this study. It was also vital to include women from rural areas, as urban-based women would probably have different circumstances than those from rural areas. Moreover, the sample included both married and single women and women both with and without children to understand the experiences of both parties and the interplay between them concerning traditional practice, identity, and norms. Although both sets of women have unique experiences within their social context, some aspects of social life are contrasting. Recognizing these differences and similarities adds to the complexity and quality of the study. Therefore, when selecting respondents for the study, the guiding principle was to ensure as much heterogeneity as possible.

For this study, in-depth conversational interviews (narratives) were proposed to “tap” the female entrepreneurs’ voices. The narrative interview is identified as an unstructured, in-depth interview with specific features (Creswell, 2007). Conceptually, the idea of narrative interviewing is encouraged by a criticism of the question-response outline of most interviews (Bauer, 1996). Bauer (1996) argues that the narrative interview should be conducted through five phases: preparation, initialisation, main narration, questioning, and concluding talk. The researcher was able to complete the 14 interviews which took place in Sri Lanka from July to November 2016. The interviews were conducted in their place of business and lasted between one and a half hours. Most of the interviews were carried out during evening sessions, except for two interviews when an alternative time was more convenient for the respondents. Furthermore, the second interview took place 1 or 2 months after the first interview. Therefore, it was possible to read and re-read the interview transcript data from the first interview, which helped identify critical issues, contradictions, inconsistencies, and evasions before going on to the second interview as a new session. Between July 2016 and November 2016, the researcher visited all female entrepreneurs selected for this study. The researcher was able to get first-hand experience of their business place surroundings and networking relationships. Moreover, observation offers an excellent opportunity to obtain detailed and authentic insights into real situations, including actions, conversations, and physical descriptions. Upon arrival at the research field, the researcher tried to develop good social relationships with both the respondents and villagers.

Thematic analysis was used to analyze the collected data. There are different styles of performing thematic analysis; however, many researchers use a five-step process: familiarisation, coding, generating themes, reviewing themes, and defining and naming themes. Considering this, the study also followed the five steps process. The first step of familiarisation involved transcribing the recorded interviews in detail for the research. Extracts were reduced where necessary to exclude unnecessary discussions, repetition, and examples provided by the participants. The next step was the coding/labelling process. Coding means highlighting sections of transcripts and using shorthand labels or “codes” to describe their content. The third stage took in identifying themes. Usually, several codes can be combined into one theme. Themes are generally broader compared to codes. Then, the researcher tried to make sure that the identified themes had value and accurate representations of the data by reviewing themes. The final stage was defining and naming the themes, and it involved coming up with a concise and an easily understandable name for each theme.

## Results and discussion

### How do female entrepreneurs form and develop their networks?—Themes derived from women’s narratives

How female entrepreneurs form and develop network relationships during their business life cycle has been the focus of interest of many researchers. The analyses of the different narratives of female entrepreneurs have produced six themes.

### Parents’ relocation due to seasonality

Tourism’s seasonality affects the distribution of domestic activities and hence the negotiation of related gender roles. Participants rely not only on help from their immediate family members, such as their partners or children but also their parents. As many of the respondents’ parents often live in different cities with their children, relocation of domestic activities occurs. The parents come to live with the female entrepreneurs for 4–6 months during the high season to help their children with household and business activities. This situation is noticeable for study respondents with small kids. My field experience evidenced this:

*I went to meet Kumudu on 13th July, 2016, and she asked me to wait for her for another few minutes; meanwhile, her mother talked to me. She said she comes to live with her daughter for a few months every high season to help her with domestic and business activities. (field notes).*

This is an example of “temporary migration” and the consequent seasonal relocation of domestic activities. Deepa is a guest house owner in Hikkaduwa whose parents (who live permanently in Baddegama) relocate to Hikkaduwa for the high season to help their daughter with domestic activities, such as childcare. Her parents take on what Deepa calls “child parking” responsibility. However, they do more than just “child parking”; they carry out various social and domestic activities, such as caring for the young children, taking the children to schools and classes, feeding them, cooking, cleaning, and paying bills on Deepa's behalf. They also provide emotional support for their daughter in the busy and stressful high season. These findings correspond with the literature. For example, Lindvert et al. (2017) highlighted the importance of household and family effects on entrepreneurship in the Pakistani culture rather than the economic benefits of entrepreneurship.

A similar trend in parental relocation from their permanent residence to provide domestic assistance is observed in Kumudu’s story, when her mother comes to help out during the high season months, relocating from her home. During the high season, as Kumudu says, domestic tasks, such as *“cleaning, the clothes, the food are all responsibilities of my mother- when she is here”*.

The extracts above show the restructuring of social and domestic duties on a seasonal basis caused by tourism, an aspect that is absent from most tourism literature (Bakas, 2014). While invisible, this tourism-related relocation of domestic duties displays how tourism encourages female entrepreneurs to negotiate their gender roles by effectively off-loading these activities onto a relocated parent. As the relocated parents are only seasonally available to take over domestic responsibilities, they temporarily disrupt negotiations of domestic activities’ gender roles by taking responsibility for their children’s domestic welfare. While this may reduce negotiations of domestic activities-sharing within the family, upon their parents’ departure, the female entrepreneurs are left to renegotiate these activities and their related gender roles. This adds complexity to how gender roles are transformed, as the tourism entrepreneurs can take up productive roles more effectively as they delegate domestic and childcare responsibilities to their parents. These findings complied with previous literature. For example, McAdam et al. (2019) revealed that women's networks might provide opportunities for mutual support and building confidence which are crucial factors for forming an entrepreneurial identity.

### Access to networks beyond local customers—a focus on international customers

These female entrepreneurs regarded relationships with international customers as part of their everyday lives. Relationship with customers was further developed through extending reciprocity and hospitality (Beriso, 2021). This reciprocity and the appreciation of relationships with international customers, such as gift-giving, free meals and socialising with customers (organised fun activities, pleasure trips), were evident throughout the narrative interviews and the researcher's fieldwork observations. Such reciprocal behaviour demonstrates these entrepreneurs' close relationships with international customers. Deepa described her experience:

*Our guests are primarily repeated customers. Most guests are British or Russian, and they call or email to make a reservation. They have trust in us and are assured of good service here. Some call this their second home.*

According to the above extract, having repeat customers is a sign of business success in relationships. Furthermore, these women thought about keeping quality relationships to make their international customers happy by providing good service and controlling their negative emotions. Santos et al. (2021) found that involvement and emotions are crucial in tourism-related businesses. Consequently, understanding emotions’ role can lead to customised emotional tourism services (Santos et al., 2021). For example, tourism marketers can develop promotional activities to match existing and potential tourists (Santos et al., 2021). The researcher’s field experience confirmed this:

*I stayed three nights in three different guesthouses. All the rooms I stayed in were arranged with care, and they are of an excellent standard. They maintain reasonable cleanliness, sanitation, quality food, etc. They mainly focus on international customers. I saw international tourists enjoy homemade local dishes during a homestay* (Field notes, 11th August 2016).

All the women confirmed their knowledge of their customers’ needs, desires, dislikes and so forth, which enabled them to keep intimate relationships with customers (Kamberidou, 2020). One of the respondents (Seethani) told her knowledge about international customers: “*What Brittan’s like is not what Americans like. Russians’ likes are different from others*”. Even though none of the women had participated in intercultural awareness training, everyone had adequate knowledge about cultural diversity and understanding of their international customers.

All the participants were aware of their cultural values, beliefs, social norms, traditions, and artefacts that appeal to international customers (Kamberidou, 2020). These women’s products and services were in line with cultural values and elements, such as art, design, and materials unique to the country. For example, Chaya (the tourist shop owner) stressed that her products are unique to Sri Lanka, and she used such uniqueness to attract customers:

*Sri Lanka’s best brand name in batik today… its mine… Customers from different countries such as Germany, Sweden, France, UK, and the USA so on come to my shop, and they are delighted with my products (Chaya). So the image is significant for repeat customers.*

To target international customers, there are souvenir shops with masks, puppets, shells, corals, batiks, paintings, and Sri Lankan tea. Jewellery is adapted to western tastes; it is not of the vulgar, golden type but the simple, silver type with beautiful semi-precious stones. The images and attitudes toward a destination formed in an individual's mind influence their visit intentions (Beriso, 2021).

### Close relationships with locals

Dramatic changes and development of the rural areas in recent years have introduced a new development model, theorised by Rae (2005) as “neo-endogenous development”, based on local development by connecting endogenous material and cultural potential (Rae, 2005). According to the “neo-endogenous” development model, social and cultural capital is the main driving force for economic development and the value of local institutions and resources and awareness of business opportunities in the same locality is considered as crucial (Bosworth & Farrel, 2011).

An example in the Sri Lankan tourism sector is maintaining healthy relationships with other local businesses, such as greengrocers, fishmongers, beach vendors, craft sellers, taxis, local authorities and the local NGO, resulting in better sales opportunities for locals and quality services for tourists. These findings complied with Baggio and Valeri (2020), as they found that to survive in the industry, entrepreneurs should develop more positive bonds with people, even their business rivals.

Hotels and restaurants focus on the cost advantages of using local labour and supplies, and guests are motivated to enjoy local activities.

*I also try to help street vendors and small businesses. So the fishmonger and the greengrocer are friendly to me (Kishani).*

Tourism business owners maintain good relationships with three-wheel drivers. When a driver takes a guest to a particular place, they usually receive extra payment. Three-wheeler taxis, or *‘Tuk Tuk’,* are vital to Sri Lanka’s transport network. They operate similar to taxis and are usually a convenient and highly cost-effective way to get around. Many foreigners find it an easy and cheap way to travel short distances during their stay, as they have an open roof, allowing viewing the surroundings, and enjoy the wind blowing by.

The hotel owner, Kishani, maintains a good relationship with the taxi drivers. The hotel allows drivers who want to register at the hotel and is limited to 15 drivers. Furthermore, the hotel provides an identity card and a uniform for drivers with the hotel logo. The Kishani Hotel does not need to interevent to decide in the charges of rides but provides the drivers and their customers a hint about a fair amount. As Kishani explains:

*This is a perfect method, and the hotel has a positive relationship with the three-wheel drivers. It is suitable for all parties. When other three-wheel drivers bring in guests, I pay them a commission.*

Some female-owned hotels in this study agree with a few three-wheel drivers waiting at the hotels. For example, Seethani hotel repainted the vehicles in the same colour to show that the vehicles are only for hotel guests. As Seethani explains:

*In the beginning, it seemed like an effective strategy, as the drivers get rides from the hotel guests, and the hotel knows with whom their guests were going. However, after complaints of charging and some other bad experiences of guests with drivers, I had to advise my guests not to use these vehicles. After that, I had to face many problems from three-wheel drivers. So, we always try to keep a good relationship with them.*

Seethani’s hotel is located in the Hikkaduwa town area, and there are many three-wheel parking areas everywhere. Therefore, the hotel guests will not essentially get a taxi from in front of the hotel. Though Kishani and Seethani’s hotels’ initiatives are examples of positive three-wheeler regulation, they provide reliability and safety with the hotel registration.

### Informal recruitment

Local relationships are essential in recruitment, especially for tourism-related businesses, depending on local resources (Bosworth, 2012). All the women in the study also mentioned that recruitment typically happens from the local community by word of mouth through personal networks, such as current staff members, fishmongers, greengrocers, etc. For many areas, seasonality continues to be an essential aspect of life, and they need staff that can fit in around this peak demand. Indrani relates:

*Every year, the employees are different because we cannot employ anyone permanently as the business does not function for half the year. We speak to the fishmonger, the man who sells vegetables or asks around for employees.*

Informal recruitment tends to be connected with low-skilled labour (Bosworth, 2012). In line with this, most women in this study actively hired individuals without good prior qualifications or relevant work experience. This method eliminated a significant barrier to recruitment, in which applicants would otherwise have to invest in capability and gain experience to meet even essential requirements. This is critical for recruiting underprivileged individuals from the local community, because creating pre-requisite qualifications for jobs (as many large tourism organisations currently involve) almost excludes more impoverished people from entree to even the lowest skilled jobs. Furthermore, employing local staff and recruiting informally is typically cheaper for the company than bringing in outsiders, which is why Sri Lankan tourism substantially reduces poverty. While jobs in the tourism industry are often considered unskilled and seasonal, the importance of income generation to local economies should not be taken lightly. This was evidenced by my fieldwork experience, as follows:

*Many employees are local, and very few trained staff members come from areas outside of Hikkaduwa. A local community labourer is paid around RS1000) daily. Trained staff members’ daily pay is 2,500.00–3,000.00 (Field notes, 9th September 2016).*

One of the most prominent challenges hotels and other tourism-related businesses faced stemmed from social issues due to local and informal recruitment. In general, employees from the local area prioritise family and community over job responsibilities, challenging employers' different expectations about ethical work behaviours, such as attendance, punctuality, and time off. Moreover, most of the staff members of these female-owned businesses do not usually have prior job experience and learning by doing was used as the only method of training. The majority of women take advantage of their so-called natural strengths, such as building relationships, and creating a culture of collaboration (Kamberidou, 2013).

### Membership in various tourism-related organisations

One of the most effective ways for an entrepreneur to build and develop weak ties is membership in various societies (Premarathna, 2002). Belonging to similar social groups, such as membership in professional groups, is essential for organisational networking. Therefore, researchers argue that such professional ties involvement is significant to small business female entrepreneurs due to their lack of resources and knowledge. In female tourism businesses establishing relationships with professional organisations like the government provides more access to resource acquisition than strong business ties (Ribeiro et al., 2021).

Hikkaduwa Tourism Service Providers’ Association (HTSPA) is a thriving organisation in Hikkaduwa. The organisation has 23 parties, including three-wheel drivers, minivan drivers, guesthouses, tourist shops, guides, restaurants, hotels, etc. The programme was introduced by the Divisional Secretarial office, because there were numerous problems reported in the sector in Hikkaduwa. The HTSPA was established to overcome such issues and ensure the safety of tourists, increase the living status, and keep sustainable tourism in the area.

I found that female entrepreneurs were members of various organisations in a few cases. For example, Kumari owns a travel and tour agency, and her business is at the developing stage. However, unlike other women, her network consists of professional ties. As Kumari explained:

*There is also a TAASL members’ night held annually, and I attend that because many agents attend that. We can make contacts and, based on these connections, we receive beachside tours and surf riding business opportunities.*

She further explained that, by taking membership in several organisations, women could develop their business networks and make contacts with different business owners who are ready to share their experiences and expertise (Alene, 2020). More specifically, they can learn from each other by providing information and tangible resources needed in the entrepreneurial process (Wenger, 1998). Participatory learning supports female entrepreneurs in reducing problems in practice, developing entrepreneurship, and reaching independence (Rao et al., 2021). This finding is consistent with Granovetter’s weak tie theory: the more weak ties business owners have, the more connected to the world we are, and the more likely we are to receive critical information about ideas and opportunities in time to respond to them.

### Attending seminars, conferences, trade fairs, and training programs

The network supports entrepreneurs in searching for opportunities (Lechner et al., 2016). Generally speaking, events such as trade fairs, conferences and training programmes bring together individuals with a common interest in technology, products or services for a specific industry. Specifically, as a start-up company seeking to get the business's name 'out there, one needs to gain influence through these trade show events to the business's advantage.

Chaya runs a tourist shop in Hikkaduwa; it has been 4 years, since she started this business, and now there is a good demand for her clothes. Chaya gave her thoughts about attending events, such as trade fairs, exhibitions, conferences, and training programmes:

*I am a member of ASMTE, and I participate in their workshops and training programmes. I am also registered with the Department of Textiles and did a course in fashion design. It was beneficial to understand how they attract customers, use new fashion, etc.*

This quotation from Chaya shows two critical thoughts. First, she related new knowledge gained (current trends in the fashion industry) to discuss the importance of participating in training programmes. Second, she understood why the new knowledge and skills were essential by participating in the training programme. Practicably in this way supported her use of her new knowledge and skills to increase her customer base, which was confirmed by the literature. For example, through collaborative relationships, entrepreneurs can share their knowledge, resources, plans, and strategies with others and design new tourism products and services (Valeri, 2016).

Another respondent, Kumari, explained her experience of participation in training in terms of her business’s development:

*After the training programme, I introduced a self-service coffee machine to the restaurant. During the training programme, we talked about our businesses and about a book we have read, a movie we have watched. So everybody can learn from each other.*

In line with the community of practice theory (Wenger, 1998), Kumari’s idea makes known the mutual exchange of participation in a community of practice. She describes her approach being changed even as she, through her participation, changed the community. In other words, participation changes the community as the community changes the participant (Wenger, 1998).

### Attending social gatherings

Another way of evolving the network is by attending social gatherings organised by various communities, forums, neighborhoods etc. The most common approach adopted by female entrepreneurs is attending religious and cultural events organised by people of the same ethnicity (Werber et al., 2014). Contacts through social gatherings have always played an influential role in business success, and those with an extensive network of contacts are usually exposed to more opportunities and better leads (Lechner et al., 2016).

When people attend a live event, such as a social gathering, they have access to other attendees. Therefore, these events are an excellent opportunity to connect, share information, learn about peers and the sector, etc. Networking is not limited to entrepreneurs during social gatherings, and these informal connections are often invaluable. Anoma related her experiences and how she tries to contact different people in social gatherings:

*I look at every social gathering as an opportunity to find out how other people think, what they do for a living, and what they know about me. Furthermore, if I can assist them, I like to do so.*

In line with Wenger’s (1998) COP theory, Anoma’s explanation refers to the importance of being mutually engaged in activities. Therefore, she is happy to share information and resources and be responsible for others.

Another respondent, Kishani, revealed that relationships with different types of people in her daily routine Influence her business’s smooth running. As she explained:

*I also try to help street vendors and small businesses. So the fishmonger and the greengrocer are friendly to me. The people in “Galwala” in Galle are thugs, but they too are friendly with me. I visit them and participate in their weddings and funerals because we need people from all walks of life, without which it would be difficult to run this business.*

This quote from Kishani illustrates the idea of emphasising the importance of informal networking, which has been invaluable to running her business. By engaging in casual conversation with others, she learns a lot about people and who they are. She finds informal networking to be a much more effective way of running the business, and mutually beneficial to a relationship. Furthermore, it illustrates how networking can arise anywhere and how one does not need to be in a professional setting to network and meet people who will support your business activities and vice versa. These findings are in line with previous research conducted on voluntary associations and social capital (Wang et al., 2021).

## Conclusion

This article provides in-depth information about how female entrepreneurs form and develop their networks, focusing on the structural dimensions of networking, and thus it contributes knowledge to the extant literature. As highlighted in the literature, many researchers focused on quantifying the network size, frequency, and density, yet little attention has been paid to analysing network actors' actual contribution and involvement in female-owned businesses. Therefore, the findings of this study are anticipated to fill a further gap in the literature. Another major theoretical contribution lies in this research connecting female entrepreneurship and networking while researching the tourism sector.

Findings suggest that, in Sri Lanka, small female businesses depend on more informal recruitment, and they have strong relationships with local communities, but they focus on customers beyond the locals. Female tourism entrepreneurs face unique challenges, such as seasonality and 14-h workdays, affecting how they negotiate the activities required to perform daily and which, inter-generationally, include household duties. Identifying a liminal gender re-negotiation period at the end and the beginning of the season, stimulating seasonal gender role negotiations, adds an exciting dimension to the perceived impacts of seasonality. While seasonality has been examined from various directions as a structural cause of disruption to economic processes, very little has been written on the effects of seasonality on women’s domestic tasks. By revealing how seasonality acts on the relationship between entrepreneurship and gender roles, this study expands the knowledge on tourism seasonality’s socio-economic impacts. Furthermore, this study reveals that female tourism entrepreneurs’ parents temporarily move location to support their children in their domestic tasks. While generally invisible within the tourism literature (Figueroa-Domecq & Segovia-Perez, 2020), this tourism-induced seasonal relocation acts as a rearrangement of household responsibilities, as female entrepreneurs off-load such tasks onto relocated parents, resulting in a temporary disruption of gendered negotiations surrounding domestic duties. In this setting, while many researchers from developed countries focus on the economic benefits of entrepreneurship, this study offers an understanding of how entrepreneurship could be used as a tool for flexibility and freedom. It also helps open eyes to more profound implications for social outcomes from networking relationships than economic ones.

The study’s findings communicate to entrepreneurs, policymakers, and supporting organisations. Over the past 20 years, small business development has been the central area of donor support for developing economies. This traditional support system has focused on providing more software (training and consulting) and hardware support (loans) for small enterprises. However, they should focus their consideration and programmes on providing increased resources and more facilities and networking input. Therefore, networking is one of the answers, even though it is not a magical solution, because through established network relationships, businesses can gain access to external resources and encouragement and influence. It was identified in the study that the interactive effects of actors of networks and resources stimulate the development of businesses, but not the resources alone. Therefore, entrepreneurial development programmes should extend support to female entrepreneurs to establish and develop effective relationships with external actors. Thus, several collective organisations of entrepreneurial women could help promote entrepreneurship among women and thereby empower them to join the economic mainstream, leading to the social development of Sri Lanka.

A few limitations are linked with this study, some of which originate in constraints on time and money. Some of these, while limitations, provide paths for future research. The data collected for this study depended on the responses from one party (an entrepreneur) and can be viewed as somewhat subjective. Preferably, a second party would validate at least part of the information collected about the entrepreneurs. Data collected from these women’s husbands, other family members, or employees could have been used to confirm the business’s information if more time and resources had been available. Moreover, this study focuses on female entrepreneurs’ networking experience in Sri Lanka, and there are various conclusions from the study. Even though some researchers suppose their findings of a single country are generalisable to other countries, these findings cannot be generalised as a global phenomenon. Similarly, some of the aspects of this research are not necessarily globally applicable, but they may be known in patriarchal societies.

## Data Availability

The data sets generated and/or analysed during the current study are not publicly available as this is qualitative research but are available from the corresponding author on reasonable request.

## Abbreviations

ASMTE: Association of Small and Medium Tourism Enterprise
COP: Community of Practice Theory
HTSPA: Hikkaduwa Tourism Service Providers’ Association
NGO: Non-Government Organizations
TAASL: Travel Agents Association Sri Lanka
UNWTO: United Nations World Tourism Organization
